# Effects of Tart Cherry Powder on Serum Uric Acid in Hyperuricemia Rat Model

**DOI:** 10.1155/2020/1454305

**Published:** 2020-07-22

**Authors:** Ruirui Li, Yuefeng Tan, Yanxia Li, Xia Zhu, Xinyao Tang, Lishi Zhang, Jinyao Chen

**Affiliations:** ^1^West China School of Public Health and West China Fourth Hospital, Sichuan University, Chengdu, China; ^2^Food Safety Monitoring and Risk Assessment Key Laboratory of Sichuan Province, Chengdu, China

## Abstract

Hyperuricemia, as a critical risk factor for various adverse clinical outcomes, shows a trend of increasing prevalence among young-aged population. Dietary adjuvant therapy by function foods, such as tart cherry, is promising. Thus, effects of tart cherry powder specialized in hyperuricemia were explored via establishing a hyperuricemia model in Sprague Dawley rats by cotreatment with oteracil potassium and adenine. The results indicated that low dose of tart cherry powder (0.17 g/kg·bw) showed effects on hyperuricemia by slightly decreasing serum uric acid and improving kidney injury, whereas high dose of tart cherry powder (0.50 g/kg·bw) could merely alleviate kidney injury. Meanwhile, adenosine deaminase activity rather than xanthine oxidase activity was affected at low dose, which reveals low dose of tarty cherry powder may be beneficial to hyperuricemia through reduction of ADA activity, and its reported potentials on antioxidation or anti-inflammation provide clues for further study.

## 1. Introduction

Hyperuricemia is a metabolic disorder diagnosed as overproduction of the serum uric acid level, that is, >357 mmol/L in females and >416 mmol/L in males [1], which pathophysiologically results from renal less excretion mainly (about 90%) [2] and overproduction of uric acid [3]. Further causes include decreased renal function, side effects of certain kinds of drugs, function impairment of some transporters facilitating renal uric acid excretion, and partly missense mutation in D-lactate dehydrogenase [4]. The overproduction of uric acid along with renal disorders results in the supersaturation of monosodium urate crystals [5] that deposit in joints and increase inflammation and causes gout [6]. Besides, hyperuricemia itself is regarded as an independent risk factor for various diseases including cardiovascular diseases [7], chronic kidney disease [8], hypertension [9], stroke [10], obesity [1], and diabetes [11] and is one of the five diagnosis indices of metabolic syndrome [12].

The prevalence of hyperuricemia dramatically increased and was higher in men than in women during the past decades. It was estimated that around 32.5 million individuals were afflicted by hyperuricemia in the United States in 2019 [13]. Besides, the socioeconomic burden originated from hyperuricemia and related diseases are countless. Thus, effective measurements and treatments should be taken to cope with it [1]. Nowadays, antihyperuricemic drugs are mainly categorized into uricostatic drugs (e.g., oxypurinol and febuxostat) and uricosuric drugs (probenecid, sulphinpyrazone, BCX4208, and pegloticase) and given singly or combinedly for lowering UA synthesis or ameliorating UA secretion [14]. However, impaired renal function may cause the retention of kidney-excreted medicines and their metabolites, which consequently prolongs their plasma half-lives and increases the risk of serious adverse events, such as Stevens–Johnson syndrome and toxic epidermal necrolysis induced by allopurinol [15]. It was estimated that patient adherence to prescribed urate-lowering therapies ranges from 20% to 70%. Besides, a survey showed that patients had a high degree of interest in nonpharmacological therapies for gout [16]. Physicians advise patients to take fruits and vegetables more in daily life as well [1]. The above proofs suggest that other additional strategies such as dietary intervention with efficacious function foods may be promising in alleviating hyperuricemia in the long run.

Recently, nutritional studies have focused more on the use of function foods to improve human health. Tart cherry (*Prunus cerasus*, sour cherry) is increasingly popular with several reported beneficial effects including lowering blood pressure, modulating blood glucose, enhancing cognitive function, protecting against oxidative stress [3], reducing inflammation, and alleviating muscle damage commonly associated with prolonged physical effort [17]. Two epidemiological studies suggested that tart cherry could decrease serum uric acid in healthy population [18] or overweight/obese adults [15]. An animal study of 2 weeks showed that tart cherry juice treatment reduced the serum uric acid levels in hyperuricemic rats in a time-dependent manner [19]. A system review revealed an association between cherry intake and a reduced risk of gout attacks [20]. These results implied the potential on improving hyperuricemia as well as exhibited a limitation that presents evidence is lacking in hyperuricemia population for a long treatment by tart cherry. Therefore, it is of interest to investigate the potential of tart cherry to improve hyperuricemia in a relatively long term. Nevertheless, whether to treat asymptomatic hyperuricemia or not is still lacking in universal agreement, and early treatment is hardly realized as a practice of preventing concept at present [21]. Hence, establishing a steady hyperuricemia rat model and exploring the benefits of tart cherry for hyperuricemia are more feasible. This study aims to explore the long-term effects of tart cherry powder on the progressive hyperuricemia and the secondary renal and liver injury comparing with classical medicine effects in hyperuricemic rats caused by oteracil potassium and adenine.

## 2. Materials and Methods

### 2.1. Materials

Tart cherry powder was provided by By-Health Co., Ltd (Zhuhai, Guangdong, China) and extracted from *Prunus cerasus* by BerryShield^TM^ technology with the proportion of 13 : 1, and total polyphenol was up to 7.4% ± 3.0 (w/w). Oteracil potassium (CAS No. 2207-75-2, purity ≥ 99.9%) was purchased from JKamaishu (Shanghai, China) biotechnology Co., Ltd. Adenine (CAS No. 73-24-5) was purchased from BioFroxx L.L.C. (Solon, OH, USA). Allopurinol (CAS No. 315-30-0) was purchased from Shanghai Xinyi Vientiane pharmaceutical Co. Ltd (Shanghai, China). Adenosine deaminase (ADA) kit and xanthine oxidase (XOD) kit were purchased from Nanjing Jiancheng Biology Engineering Institute (Nanjing, Jiangsu, China). Sodium carboxymethylcellulose (CAS No. 9085-26-1) was purchased from Chengdu Kelong chemical reagent factory (Chengdu, Sichuan, China).

### 2.2. Methods

#### 2.2.1. Animals

SPF-grade male Sprague Dawley (SD) rats (body weight range: 200∼250 g) were purchased from Dashuo Laboratory Animal Reproduction Center (Chengdu, Sichuan, China) (Certificate No. SCXK2013-24). The rats were kept at the Animal Laboratory Center of West China School of Public Health (Chengdu, Sichuan, China) (Certificate No. SCXK2018-011). All animals were maintained in controlled animal care facility with 12 hr day and night cycle with controlled temperature (20–25°C) and humidity (40–70%) with standard diet and water ad libitum. The animals were acclimatized for 7 days before treatment. Standard ethical guidelines of the Ethical Committee for Research on Laboratory Animals of Sichuan University were followed.

#### 2.2.2. Experimental Design

Serum uric acid was measured at day 1 for excluding those rats of serum uric acid values out of range of 95% baseline ($\bar{x}$ ± 1.96 S) [17]. Eligible rats were randomized to 5 groups (*n* = 9 in each group) including vehicle control group (*C*), model group (*M*), positive group (*P*), low-dose group (*L*), and high-dose group (*H*) randomly according to the serum uric acid of day 1. Based on the previous study on establishing hyperuricemia model in our lab [22], adenine with 5% sodium carboxymethylcellulose suspension (50 mg/kg·bw) and oteracil potassium (1.50 g/kg·bw) with 5% sodium carboxymethylcellulose suspension were given to the animals of model group, positive group, and two experimental groups at 9 : 00. Meanwhile, 5% sodium carboxymethylcellulose solution was given to the vehicle control group. After 5 h, treatments were administrated: pure water was given to the animals of vehicle control group and model group, allopurinol solution (27.0 mg/kg·bw) was given to the animals of positive group animals, and two experimental groups were separately given tart cherry powder suspension at the doses of 0.17 g/kg·bw and 0.50 g/kg·bw which extrapolated from 10 times and 30 times of recommended human intake (1 g/kg·bw/d). Above reagents were all administrated by gavage at a volume of 5 mL/kg·bw once a day for continuously 45 days (days 1–45). Details can be seen in Table 1. Body weight was measured and recorded once a week until animals were necropsied.

#### 2.2.3. Blood Collection and Measurement

Peripheral blood was collected on days 1, 15, 30, and 45. On days 1, 15, and 30, blood was obtained from orbital venous plexus after treating 2 h. At day 45, all animals were sacrificed after treating 2 h, and the abdominal aorta blood samples were collected. After centrifugation at 1300 g for 10 min, the serum was isolated and stored at −80°C for following detection. Serum uric acid, creatinine (Cr), blood urea nitrogen (BUN), aspartate aminotransferase (AST), and alanine aminotransferase (ALT) were all determined by an autoanalyzer (Olympus AU400, Japan). ADA and XOD activities were determined by using the ADA kit and XOD kit.

#### 2.2.4. Necropsy and Histopathology Analysis

The animals were anesthetized, the blood samples were collected, the livers and kidneys were excised and weighed, and liver coefficient was calculated according to the formula: liver coefficient = (liver weight/body weight) × 100%. Kidney coefficient was calculated in the same way. The liver tissue in the middle of the hepatic lobe and left kidney were fixed in 10% buffered formalin, embedded in paraffin and stained with hematoxylin and eosin (H&E) and observed under a photo microscope (Olympus DP73, Japan). The images of whole tissue sections from all animals were captured under × 200 optical magnification. Liver and kidney histological injury evaluation depended on two previous semiquantitative scoring systems. In brief, kidney injury score (range: 0∼12) was added up the scores of the following four parts, namely, tubular atrophy (0, normal tubules; 1, rare single atrophic tubule; 2, several clusters of atrophic tubules; 3, massive atrophy), tubular necrosis (0, normal tubules; 1, rare single necrotic tubule; 2, several clusters of necrotic tubules; 3, massive necrosis), lymphocytic infiltrates (0, absent; 1, few scattered cells; 2, groups of lymphocytes; 3, widespread infiltrate), and interstitial fibrosis (0, absent; 1, minimal fibrosis with slight thickening of the tubular basal membrane; 2, moderate fibrosis with focal enlargement of the interstitium; 3, severe fibrosis with confluent fibrotic areas) [23]. Similarly, liver injury score (range: 0∼8) was defined as the sum of the scores for steatosis (0, <5%; 1, 5%∼33%; 2, >33%∼66%; 3, >66%), lobular inflammation (0, no foci; 1, 2 foci; 3, 2∼4 foci; 3, 4 foci), and ballooning (0, none; 1, few; 2, many) [24].

### 2.3. Statistical Analysis

Statistical analysis was performed using SPSS 21.0 (IBM Corporation, Armonk, NY, USA). The level of significance was set at *α* = 0.05. *α* < 0.05 means that it is reasonable to regard as existing statistical difference. Normality test was conducted at first. Quantitative data satisfied with normal distribution exhibited arithmetical mean with standard deviation (x ± SD), and others were expressed by median with percentiles (M ± interquartile range). One-way variance analysis (ANOVA) was conducted to analyze the difference among 5 groups under the precondition of normal contribution and equal variance. Once difference is noted, the SNK test was used to compare the vehicle control group with the experiment group. These data, unsatisfied with normal distribution, were considered to take variable transformation to meet above prerequisite. If not, the rank sum test was used to analyze this part and ranked data.

## 3. Results

### 3.1. General Behaviors, Body Weights, and Organ Coefficients

**Figure 1:**
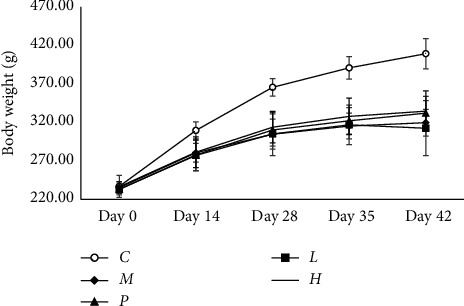
Body weight of all group animals during the experiment. Values were expressed as mean ± SE. *n* = 9 per group. *C*, vehicle control group; *M*, model group; *P*, positive group; *H*, high-dose group; *L*, low-dose group.

**Figure 2:**
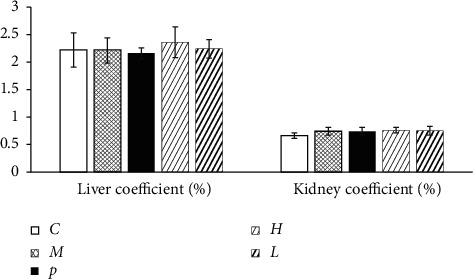
Liver coefficient and kidney coefficient of all group animals. Values were expressed as mean ± SE. *n* = 9 per group. *C*, vehicle control group; *M*, model group; *P*, positive group; *H*, high-dose group; *L*, low-dose group.

As illustrated in Figures 1 and 2, none exhibited abnormal general behaviors and difference of organ coefficients due to any intervention during the experiment. Significant decrease in body weights was noted in the model group compared with the vehicle control group starting on day 14 (*P* < 0.05).

### 3.2. Tart Cherry Powder Effects on Liver and Renal Function

#### 3.2.1. AST and ALT

No difference of AST and ALT in peripheral blood was noted among all groups after treating 45 days (Table 2).

#### 3.2.2. Serum Cr

Serum Cr reflecting glomerular function had been detected via peripheral blood in four time points, and serum Cr of the model group on day 15 was higher than that in the vehicle control group (Table 3).

#### 3.2.3. Serum BUN

As shown in Table 4, comparing with that in the control group, serum BUN in the model group is higher starting on day 15 compared with those in the vehicle control group (*P* < 0.05). On day 15, BUN in the high-dose group was lower than that in the model group. On day 45, BUN in low-dose group was lower than that in the model group.

### 3.3. Serum Uric Acid

**Figure 3:**
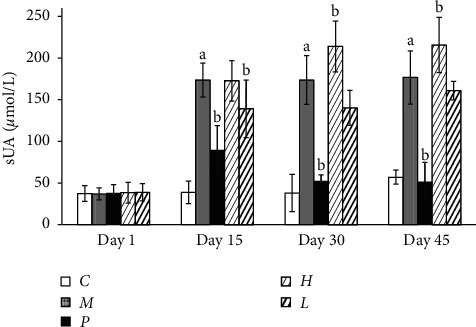
Serum uric acid of all groups at four time points. Values were expressed as mean ± SE, *n* = 9 per group. “a” denotes statistical significance (*P* < 0.05) from the vehicle control group. “b” denotes statistical significance (*P* < 0.05) from the model group. *C*: vehicle control group; *M*: model group; *P*: positive group; *H:* high-dose group; *L*: low-dose group.

As illustrated in Figure 3 and Table 5, at the beginning of the study, serum uric acid evenly distributed in the range of $\bar{x}$ ± 1.96 S. The serum uric acid increased steadily in the model group during days 1–45 and as high as four times of serum uric acid in the vehicle control group (*P* < 0.05), which demonstrated the successful establishment of hyperuricemia rat model over one month. Serum uric acid in the positive group was decreased by around 83.39∼125.10 *µ*mol/L in three time points compared with those in the model group (*P* < 0.05). Besides, there is no association between serum uric acid and time points in each group (*P* > 0.05), reflecting that serum uric acid may rarely change over time; in other words, stable serum uric acid in this experimental scheme is suitable to evaluate the efficiency of uric acid-lowering agents.

Lower serum uric acid was exhibited during the treatment in the low-dose group rather than the high-dose group compared with the model group although statistical difference was observed merely in the low-dose group on day 15 (*P* < 0.05). This result implied low-dose tart cherry powder has limited improving effect on serum uric acid.

### 3.4. Activity of XOD and ADA

Activity of XOD and ADA in peripheral blood reflected the serum uric acid metabolism. As described in Table 6, XOD and ADA activities of peripheral blood in the positive group were higher than the values in the model group (*P* < 0.05). The results reflected that allopurinol can reduce the increased activity of XOD and ADA caused by oteracil potassium and adenine. As for the treatment of tart cherry powder, ADA was decreased by tart cherry powder at the dose of 0.17 g/kg·bw (*P* < 0.05).

### 3.5. Histopathology

#### 3.5.1. Renal Histopathology

**Figure 4:**
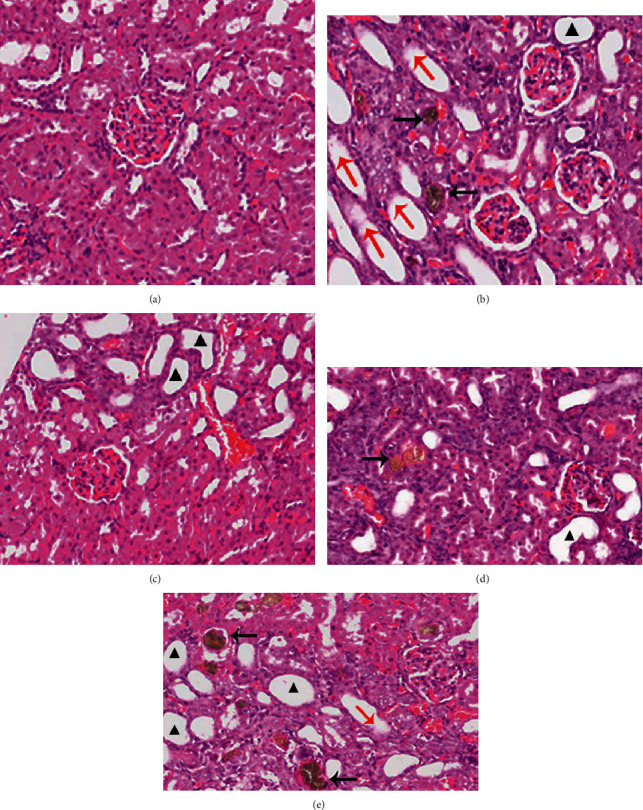
Hematoxylin and eosin stains of kidney sections of all groups (magnification, ×200). Notes: the black arrows represent brown uric acid crystal; the red arrows represent dilated tubules with necrosis and exfoliation of the tubule epithelium; and triangles label dilated tubules; (a) vehicle control group; (b) model group; (c) positive group; (d) low-dose group; (e) high-dose group.

As illustrated in Figure 4, enlargement of pale kidney was observed in all groups except the vehicle control group. The renal lesions of model group were in line with the renal lesions model of hyperuricemia (Figure 4(b)) and exhibited as the renal glomerular volume and number decreased moderately/severely, brown crystalline deposited in the tubes, and most of the renal tubules and collecting tubules dilated moderately or severely, with necrosis and exfoliation of the tubule epithelium.

In the positive group (Figure 4(c)), renal lesions were lighter than that in the model group, there were a mild/moderate decrease in glomerular volume, mild tubules dilation, and mild inflammatory cell infiltration in the interstitium. In the low- and high-dose groups of tart cherry powder (Figures 4(d) and 4(e)), the glomerular volume and number of glomeruli were reduced moderately/severely, brown crystalline was deposited in some renal tubules, and few or moderate inflammatory cells were observed in the renal interstitium. Necrosis and exfoliation of tubule epithelium coexisted with new tubule epithelium.

The frequency distribution of kidney pathology score (Table 7) in the model group was higher than that in the vehicle control group (*P* < 0.05), which reflected the oteracil potassium (1.50 g/kg·bw) and adenine (50 mg/kg·bw) caused damage on renal function. Compared with that in the model group, the lower kidney pathology score of the positive group demonstrated that treatment by allopurinol can promote renal injury to restore (*P* < 0.05). Tart cherry powder can improve the kidney function according to the image of kidney pathology, and the kidney pathology scores of high-dose group and low-dose group are also lower than that in the model groups, but without statistical significance.

#### 3.5.2. Liver Histopathology

The liver structure of the rats in the all animals was normal, as shown in Table 7 (figure of liver normal pathology has not been shown), without definite crystallization, lipidosis, edema, and liver cell necrosis.

## 4. Discussion

This study aimed to answer the question of whether tart cherry has a beneficial effect on hyperuricemia in a relatively long term with hyperuricemia rats caused by oteracil potassium and adenine. The hyperuricemia rat model is closed to human hyperuricemia pathogenesis. Oteracil potassium, as a kind of potassium oxalate, is an uricase inhibitor, which can inhibit uricase activity in rodents [25]. Adenine is a precursor of uric acid and can promote the production of uric acid in animals. Kidney injury in this model was caused by the separation and deposition of urate crystals, which was similar to the renal injury of primary hyperuricemia implicated in pathological states such as gout and inherited purine disorders. Steady increase in serum uric acid in the model group manifested the model could be used in long-term study of hyperuricemia. One limitation is that the pathogenesis of renal injury resulting from the intervention of modeling drugs (i.e., oteracil potassium and adenine) directly could not be decided.

Our study demonstrates the tart cherry powder at low dose showed limited protecting effects on hyperuricemia by slightly decreasing serum uric acid at the early stage of hyperuricemia and improving kidney injury. A latest population study demonstrated that tart cherry concentrate dose was lacking in effect on serum urate in people with gout [26]. Nevertheless, beneficial effects have been reported in previous studies. In 1950, Blau et al. demonstrated that fresh and canned cherries reduced serum uric acid concentrations in patients (*n* = 12) with gout, which is the first report of cherry's effect on serum uric acid in population [27]. Another study reported that serum uric acid was decreased by 14% from 214.1 *μ*M (3.6 mg/dL) to 178.5 *μ*M (3.0 mg/dL), in healthy women (*n* = 10), consuming 2 servings (280 g) of cherries after an overnight fast [18]. Similar results with a significant 19.4% reduction in serum uric acid were observed in 26 overweight and obese participants who consumed 240 mL/d (8 oz/d) of tart cherry juice for 4 wk [15]. It was known that (1) adenosine can be transformed into inosine by ADA and then produced hypoxanthine via nucleoside phosphorylase [28] and (2) uric acid can be converted by XOD from purines (xanthine and hypoxanthine) in the liver and distributed into blood and kidneys [29]. XOD and ADA activities in peripheral blood were also detected in this study. The results showed that ADA activity was decreased to the value in the model group at the dose of 0.17 g/kg tart cherry powder, which hinted that tart cherry may affect the activity of ADA rather than XOD activity. Within this study, serum uric acid was not decreased by high-dose tart cherry. Thus, the benefits of tart cherry decreasing serum uric acid might be limited, which indicated that tart cherry may benefit to reduce serum uric acid in early hyperuricemia but cannot replace medical therapy [3]. Besides, ALT, AST, and liver pathology were evaluated. As a result, all interventions made no difference in any groups, which mainly suggested allopurinol and tart cherry do not cause liver injury for the hyperuricemia rat model at the dose in the study.

Even if the effects of tart cherry on hyperuricemia are limited, other benefits were observed in our results which are in accordance with other studies. Improvement of pathological injury of kidney was observed after being treated by tart cherry for consecutive 45 days. In the hyperuricemia model caused by adenine and oteracil potassium, renal injury referred to the following two aspects. On the one hand, hyperuricemia can induce excessive uric acid deposition in renal interstitium, and the uric acid crystal brings about ductal arteriole smooth muscle proliferation and lumen stenosis followed by glomerular and postbulbar circulatory ischemia; meanwhile, free uric acid is related to chronic interstitial nephritis and fibrosis secondary to local inflammation, the two pathways both cause renal injury exhibited as renal tubular dermal cell dysfunction, renal hemodynamic changes, and glomerular hypertrophy and pathologic change. On the other hand, adenine can be transformed into xanthine depositing in renal tubules and lead to excessive production of oxidative radicals and lipid peroxidation, which results in renal injury [8]. It is reported that tart cherry shows the potential of antioxidation [30] and anti-inflammation [31], while the content of carotenoids and phenolics greatly contributes to its antioxidative properties. The tart cherry contained 7.4 ± 3.0 (w/w) total polyphenol detected by the MG-F-C assay in our study. In addition, antioxidant melatonin (*N*-acetyl-5-methoxytryptamine) had been reported in high levels in tart cherries [17]. Besides, tart cherry has the potential to alleviate inflammation related to hyperuricemia and gout [32]. These suggested that tart cherry powder is more likely to improve kidney injury by antioxidative or anti-inflammatory effects rather than simply reducing uric acid. Thus, reduction of uric acid by tart cherry may be beneficial for the improvement of renal injury at low dose, while the potential mechanism of antioxidation or anti-inflammation to improve renal injury should be further explored.

## 5. Conclusion

Based on hyperuricemia rats model induced by oteracil potassium and adenine, this study demonstrated limited benefits of tart cherry on hyperuricemia, which reflected reduction of serum uric acid at low dose and alleviation of kidney injury, and the reduction of serum uric acid may be related to the ADA activity rather than XOD activity. Additionally, all interventions did not cause any liver injury in this study. Further research is needed for exploring its underlying mechanism.

## Figures and Tables

**Table 1:** Animal grouping and treatment.

Group	9 : 00 reagents and doses	14 : 00 reagents and doses
Vehicle control group (*C*)	5% sodium carboxymethylcellulose	Pure water
Model group (*M*)	Oteracil potassium 1.50 g/kg·bw + adenine 50 mg/kg·bw	Pure water
Positive group (*P*)	Oteracil potassium 1.50 g/kg·bw + adenine 50 mg/kg·bw	Allopurinol 27.0 mg/kg·bw
High-dose group (*H*)	Oteracil potassium 1.50 g/kg·bw + adenine 50 mg/kg·bw	Tart cherry powder 0.50 g/kg·bw
Low-dose group (*L*)	Oteracil potassium 1.50 g/kg·bw + adenine 50 mg/kg·bw	Tart cherry powder 0.17 g/kg·bw

**Table 2:** AST and ALT in peripheral blood ($\bar{x}$ ± SD).

Group	*n*	ALT (U/L)	AST (U/L)
*C*	9	39.38 ± 10.76	139.20 ± 13.85
*M*	9	38.83 ± 8.98	135.50 ± 32.46
*P*	9	39.70 ± 14.03	145.60 ± 52.84
*H*	9	40.60 ± 10.43	149.30 ± 31.41
*L*	9	33.09 ± 8.10	110.45 ± 25.13

**Table 3:** Serum creatinine of all animals in four time points ($\bar{x}$ ± SD).

Group	*n*	Serum creatinine (*µ*mol/L)			
		Day 1	Day 15	Day 30	Day 45
*C*	9	45.17 ± 1.81	40.74 ± 2.43	46.40 ± 7.54	68.71 ± 6.27
*M*	9	46.27 ± 5.04	46.47 ± 3.82^a^	46.33 ± 2.28	70.63 ± 8.00
*P*	9	47.72 ± 3.02	46.69 ± 9.23	44.86 ± 4.56	67.65 ± 7.61
*H*	9	46.83 ± 3.78	41.97 ± 3.54	47.63 ± 5.22	69.82 ± 8.89
*L*	9	45.62 ± 3.07	41.46 ± 1.56	46.27 ± 5.43	65.42 ± 6.24

^a^Compared with the values in the vehicle control group, *P* < 0.05.

**Table 4:** Blood urea nitrogen of all animals in four time points ($\bar{x}$ ± SD).

Group	*n*	Blood urea nitrogen (*µ*mol/L)			
		Day 1	Day 15	Day 30	Day 45
*C*	9	4.13 ± 0.62	4.54 ± 0.39	6.24 ± 0.78	7.12 ± 0.69
*M*	9	4.24 ± 0.59	8.04 ± 0.81^a^	8.77 ± 1.20^a^	11.30 ± 0.81^a^
*P*	9	4.10 ± 0.61	7.91 ± 0.94	7.96 ± 1.66	10.63 ± 0.82
*H*	9	3.89 ± 0.41	6.51 ± 1.05^b^	8.80 ± 1.12	11.20 ± 1.47
*L*	9	3.84 ± 0.47	7.18 ± 0.90	8.37 ± 0.84	9.31 ± 1.45^b^

^a^Compared with the values in the vehicle control group, *P* < 0.05; ^b^compared with the values in the model group, *P* < 0.05.

**Table 5:** Serum uric acid of five groups ($\bar{x}$ ± SD).

Group	*n*	Serum uric acid (*µ*mol/L)			
		Day 1	Day 15	Day 30	Day 45
*C*	9	37.41 ± 9.43	38.72 ± 13.54	38.05 ± 22.24	57.00 ± 8.43
*M*	9	36.91 ± 7.23	173.61 ± 20.38^a^	173.62 ± 29.36^a^	176.70 ± 31.86^a^
*P*	9	38.23 ± 10.00	89.68 ± 29.26^b^	52.53 ± 7.30^b^	51.60 ± 23.11^b^
*H*	9	38.46 ± 12.41	172.67 ± 24.36	213.88 ± 30.47^b^	215.54 ± 33.10^b^
*L*	9	38.90 ± 10.34	139.08 ± 34.46^b^	140.10 ± 20.97	160.82 ± 11.05

^a^Compared with the values in the vehicle control group, *P* < 0.05; ^b^compared with the values in the model group, *P* < 0.05.

**Table 6:** The activity of XOD and ADA in peripheral blood ($\bar{x}$±SD).

Group	*n*	XOD	ADA
*C*	9	13.49 ± 1.51	16.17 ± 2.74
*M*	9	18.22 ± 1.26^a^	23.96 ± 2.43^a^
*P*	9	4.05 ± 0.84^b^	16.12 ± 2.50^b^
*H*	9	18.72 ± 1.88	20.35 ± 2.20
*L*	9	18.31 ± 1.72	13.45 ± 1.40^b^

^a^Compared with the vehicle control group, *P* < 0.05;^b^compared with the model group, *P* < 0.05.

**Table 7:** The median score of rat kidney and liver pathology (M ± interquartile range).

Group	*n*	Median score of rat kidney pathology	Median score of rat liver pathology
*C*	9	0.00 (0.00, 0.00)	0.00 (0.00, 0.05)
*M*	9	3.00 (2.00, 3.00)^a^	1.00 (0.00, 1.00)
*P*	9	1.00 (0.00, 2.00)^b^	0.00 (0.00, 0.25)
*H*	9	2.00 (0.00, 2.50)	1.00 (0.00, 1.00)
*L*	9	2.00 (1.00, 3.00)	0.00 (0.00, 1.00)

^a^Compared with the vehicle control group, *P* < 0.05;^b^compared with the model group, *P* < 0.05; *M* ± interquartile range.

## Data Availability

All the data related to this article are available from the corresponding author upon reasonable request.
